# Targeting EFNA1 suppresses tumor progression via the cMYC-modulated cell cycle and autophagy in esophageal squamous cell carcinoma

**DOI:** 10.1007/s12672-023-00664-9

**Published:** 2023-05-09

**Authors:** Houxiang Jiang, Shaoxiang Wang, Ying Liu, Chaopan Zheng, Lipeng Chen, Kai Zheng, Zhenyu Xu, Yong Dai, Hongtao Jin, Zhiqiang Cheng, Chang Zou, Li Fu, Kaisheng Liu, Xiaoshi Ma

**Affiliations:** 1 Department of Gastrointestinal Surgery, The First Affiliated Hospital of Wannan Medical College (Yijishan Hospital of Wannan Medical College), Wuhu, 241001 Anhui China; 2 Anhui Province Clinical Research Center for Critical Respiratory Medicine, Wuhu, 241001 Anhui China; 3 School of Pharmaceutical Sciences, Health Science Center, Shenzhen University, Shenzhen, 518060 China; 4 Guangdong Provincial Clinical Research Center for Geriatrics, Shenzhen Clinical Research Center for Geriatrics, Shenzhen People’s Hospital (The Second Clinical Medical College of Jinan University), Shenzhen, 518020 Guangdong China; 5 Department of Otolaryngology, Shenzhen People’s Hospital (The Second Clinical Medical College of Jinan University), Shenzhen, 518020 Guangdong China; 6 Precision Medicine Center, The First Affiliated Hospital of Wannan Medical College (Yijishan Hospital of Wannan Medical College), Wuhu, 241001 Anhui China; 7 Department of Pathology, Shenzhen People’s Hospital (The Second Clinical Medical College of Jinan University), Shenzhen, 518020 Guangdong China; 8 School of Medicine, Life and Health Sciences, The Chinese University of Hong Kong, Shenzhen, 518172 Guangdong China; 9 Department of Pharmacology, Shenzhen University School of Medicine, Shenzhen, 518060 Guangdong China; 10 Department of Urology, Shenzhen People’s Hospital (The Second Clinical Medical College of Jinan University), Shenzhen, 518020 Guangdong China

**Keywords:** EFNA1, Esophageal squamous cell carcinoma, cMYC, Autophagy, Salvianolic acid A

## Abstract

**Purpose:**

Esophageal squamous cell carcinoma (ESCC) remains one of the most common causes of cancer death due to the lack of effective therapeutic options. New targets and the targeted drugs are required to be identified and developed.

**Methods:**

Highly expressed genes in ESCA were identified using the edgeR package from public datasets. Immunostaining assay verified the high expression level of EFNA1 in ESCC. CCK-8, colony formation and wound healing assays were performed to examine the role of *EFNA1* and *EPHA2* in ESCC progression. Cell cycle was analyzed by flow cytometry and autophagy activation was determined by autophagolysosome formation using transmission electron microscopy. The small molecule targeting to EFNA1 was identified by molecular docking and the anti-tumor effects were verified by in vitro and in vivo models with radiation treatment.

**Results:**

*EFNA1* was highly expressed in esophageal cancer and significantly associated with poor prognosis. Downregulation of *EFNA1* remarkably inhibited cell proliferation and migration. Furthermore, decreased *EFNA1* significantly suppressed the expression of cMYC along with its representative downstream genes involved in cell cycle, and activated autophagy. Similar effects on ESCC progression were obtained from knockdown of the corresponding receptor, EPHA2. The potential small molecule targeting to EFNA1, salvianolic acid A (SAA), could significantly suppress ESCC progression and increase the sensitivity to radiotherapy.

**Conclusion:**

We revealed that *EFNA1* facilitated the ESCC progression via the possible mechanism of activating cMYC-modulated cell proliferation and suppressing autophagy, and identified SAA as a potential drug targeting EFNA1, providing new options for the future treatments for ESCC patients.

**Supplementary Information:**

The online version contains supplementary material available at 10.1007/s12672-023-00664-9.

## Introduction

Esophageal cancer (ESCA) is the 7th most frequently diagnosed malignant carcinoma and the 6th leading cause of cancer-related death worldwide [1]. It is mainly consisted of two histological subtypes, the esophageal adenocarcinoma and esophageal squamous cell carcinoma (ESCC). Approximately 90% of ESCA patients will be further diagnosed as ESCC, a more aggressive and lethal type with only 26.2% of 5-year postoperative survival rate [2]. Although patients could get benefits from the development of current treatments like surgical resection, radiotherapy and chemotherapy, the outcomes are still unsatisfactory [3]. Plus, the preoperative cisplatin and 5-fluorouracil-based chemotherapy or radiotherapy for down-stage the disease usually bring severe side effects for the patients, even would cause a delay in surgery for patients who respond poorly [4, 5].

With the advances in high-throughput sequencing technologies, critical molecules involved in ESCC progression have been unveiled gradually, including *EGFR*, *HER-2*, *VEGFR* as well as some epigenetic molecules [6–8]. The targeted drugs have soon been developed and proven effective to improve the prognosis of ESCC patients at different levels [9–11]. Despite of the progress we have achieved in recent years, unstable efficacies, drug resistance for patients with mutations in related genes or severe adverse effects have limited the applications of these target drugs [12]. New targets and the targeted drugs are required to be identified and developed to overcome these limitations.

EFNA1 is a member of ephrin family which has been reported to be involved in multiple malignant progresses like tumor growth, invasiveness and metastasis [13–15]. In hepatocellular carcinoma, Iida et al. showed that increased expression of *EFNA1* could significantly promoted tumor growth by the potential downregulation of cell cycle inhibitor p21 and upregulation of angiogenesis factor angiopoietin 1 [16]. In mesothelioma, *EFNA1* could suppress the tumor growth via the induction of miR-320b-regulated apoptosis and the miRNA let-7-mediated repression of the RAS expression [17, 18]. However, the exact role of *EFNA1* in ESCC progression and the potential value in ESCC treatment have barely been explored and remain unknown.

Here, we integrated different public datasets and identified *EFNA1* with the most significant correlations with ESCA progression and dismal prognosis. Knockdown of *EFNA1* significantly suppressed proliferation and migration of ESCC cells under the potential regulation of cMYC and autophagy signaling pathways. Salvianolic acid A (SAA), screened as a small molecular medicine targeting EFNA1 via molecular docking, showed significant inhibitory effects on ESCC cell proliferation and tumor growth. The additional SAA treatment upon radiation remarkably increased the sensitivity of ESCC to radiation therapy, suggesting a novel therapeutic option for ESCC in future administrations.

## Materials and methods

### Expression analysis of ESCA patients

RNA-seq profiles and clinical data of 160 patients with ESCA were obtained from the TCGA database. Differentially expressed genes (DEGs) were identified using the edgeR package and filtered by fold change > 2, *p* value < 0.05 and false discovery rate < 0.05. They were further validated in four other gene expression omnibus datasets including GSE23400, GSE29001, GSE33426 and GSE38129. The overlapped genes were considered as potentially critical genes in ESCA progression.

### Immunostaining

The human ESCC tissue microarray (HEsoS180Su07, Shanghai Outdo Biotech Co. Ltd., China), consisted of 63 cancerous tissues and the corresponding normal tissues, was used to examine the expression characteristic of *EFNA1*. Immunostaining assay was performed as described by Yu et al. (2013) using the anti-EFNA1 antibodies (diluted at 1:200, Abcam, cat. no. ab238505) and the HRP-conjugated secondary antibody (diluted at 1:2000, Abcam, cat. no. ab7090). The EFNA1 staining score was evaluated by two qualified pathologists based on the staining rate and intensity.

### Construction of *EFNA1* knockdown cells with shRNA

Two shRNAs were used to suppress the expression of *EFNA1* in ESCC cell lines (Additional file 4: Table S1). The non-targeting scrambled shRNA-NC contained a sequence with no significant homology to any known human gene (HANBIO Co. Ltd., China). Virus packaging and cell transfection were performed as described previously [19].

### qRT-PCR

Total RNA was extracted using the RNeasy Mini Kit (QIAGEN GmbH, cat. no. 74104, Germany) according to manufacturer’s instructions. The cDNA synthesis was performed using a PrimeScript RT Master Mix kit (Takara, China) according to manufacturer’s instruction. qRT-PCR was performed using the TB Green Premix EX Taq II system with a Roche LightCycler 96 qPCR instrument (Roche Diagnostics, Germany). The primer sequences used in this study were listed in Additional file 5: Table S2. Gene relative expression levels were normalized to *GAPDH* expression and analyzed using the 2^−ΔΔCt^ method.

### Western blot

The expression levels of candidate proteins were examined by western blot analysis as previously described [20]. The antibodies were anti-β-actin (diluted at 1:1000, CST, cat. no. 3700S), anti-GAPDH (diluted at 1:1000, CST, cat. no. 5174S), anti-AKT (diluted at 1:1000, CST, cat. no. 4691S), anti-p-AKT (diluted at 1:1000, CST, cat. no. 4060S), anti-p-CDC2 (diluted at 1:1000, CST, cat. no. 4539S), anti-cMYC (diluted at 1:1000, CST, cat. no. 2983S), anti-cyclin D1 (diluted at 1:1000, CST, cat. no. 55506S), anti-EFNA1 (diluted at 1:1000, Abcam, cat. no. ab124911), anti-EPHA2 (diluted at 1:1000, Abcam, cat. no. ab273118), anti-LC3 (diluted at 1:1000, CST, cat. no. 12741 T), anti-mTOR (diluted at 1:1000, CST, cat. no. 2983S), anti-p-mTOR (diluted at 1:1000, CST, cat. no. 5536S), anti-p62 (diluted at 1:1000, CST, cat. no. 39749S), anti-p70S6k (diluted at 1:1000, CST, cat. no. 97596S), anti-Rb (diluted at 1:1000, CST, cat. no. 9309S), and anti-p-Rb (diluted at 1:1000, CST, cat. no. 8516S). Expression level of each protein was examined three times.

### Cell proliferation and migration assays

KYSE30 and KYSE450 cells with or without *EFNA1* downregulation were seeded into plates or dishes at an appropriate density. Cell proliferation was assessed by Cell Counting Kit-8 (CCK-8) and colony formation assay at indicated time according to previously described [19]. Cell migration was evaluated by wound healing assay as described elsewhere except that cells were cultured in a FBS-free medium for 24 h and then imaged [21].

### Cell cycle analysis

KYSE30 and KYSE450 cells were seeded into a 6-well plate at a density of 3 × 10^5^ cells/well and cultured at 37 °C with 5% CO_2_ for 2 days. After harvested by trypsinization, they were fixed with 70% ethanol at 4 °C overnight. Cells were subsequently washed by precooled PBS and incubated with 100 μg/ml RNase A and 50 μg/ml propidium iodid (PI) staining solution for 30 min at 4 °C. Then they were immediately analyzed by flow cytometry.

### Transmission electron microscopy

ESCC cells were collected and centrifuged at 600 g for 5 min. The pellet was washed and stored in 70% Karnovsky’s fixative at 4 °C. Samples were processed and embedded under standard protocols. The ultrathin sections were viewed and photographed using a Hitachi 7000 transmission electron microscope (Tokyo, Japan).

### Molecular docking

The three-dimensional structures of 1900 candidate molecules from the MedChemExpress FDA-Approved Drug Library Plus were output using the LigPrep Module of Schrodinger software. We screened for the candidate molecules that potentially bind to EFNA1 by Maestro interface (version 11.4) of Schrodinger software and drew the three-dimensional interaction diagram using PyMol. Top 50 molecules were selected and 45 of them were purchased from MedChemExpress (NJ, USA).

### Animal treatment

The female BALB/c nude mice with 6 weeks of age and 18–22 g weight were obtained from the Guangdong Medical Laboratory Animal Center (Guangdong, China). All mice were housed at 20 ± 2ºC and 55 ± 5% relative humidity, with ad libitum access to commercially available food and water throughout the experiment.

KYSE450 cells with or without *EFNA1* knockdown were injected subcutaneously into mice with the concentration of 1 × 10^7^ cells/mice, respectively. After 21 days, mice were sacrificed and sampled. The tumors were then measured and weighed to evaluate tumor growth.

The tumor xenografts mice constructed with KYSE450 cells were randomly assigned to four groups as NC, SAA, 5GY and SAA + 5GY. Mice from each group received 100 μl PBS/day orally, 100 μl SAA (20 mg/kg/day) orally, 100 μl PBS/day orally plus 5GY radiation in a single session, and 100 μl SAA (20 mg/kg/day) orally plus 5GY radiation in a single session, respectively. After 21 days the animals were sacrificed and sampled. The tumors were then measured and weighed to evaluate tumor growth.

### Statistical analysis

All values were presented as means ± standard deviation unless indicated otherwise. Data were analyzed by one-way or two-way analysis of variance (ANOVA) and differences between groups were evaluated using the Student-Newman-Keuls multiple comparison test in SPSS version 20.0. Differences were considered statistically significant at *p* < 0.05.

## Results

### *EFNA1* was highly expressed in ESCA and significantly correlated with tumor progression

**Fig. 1:**
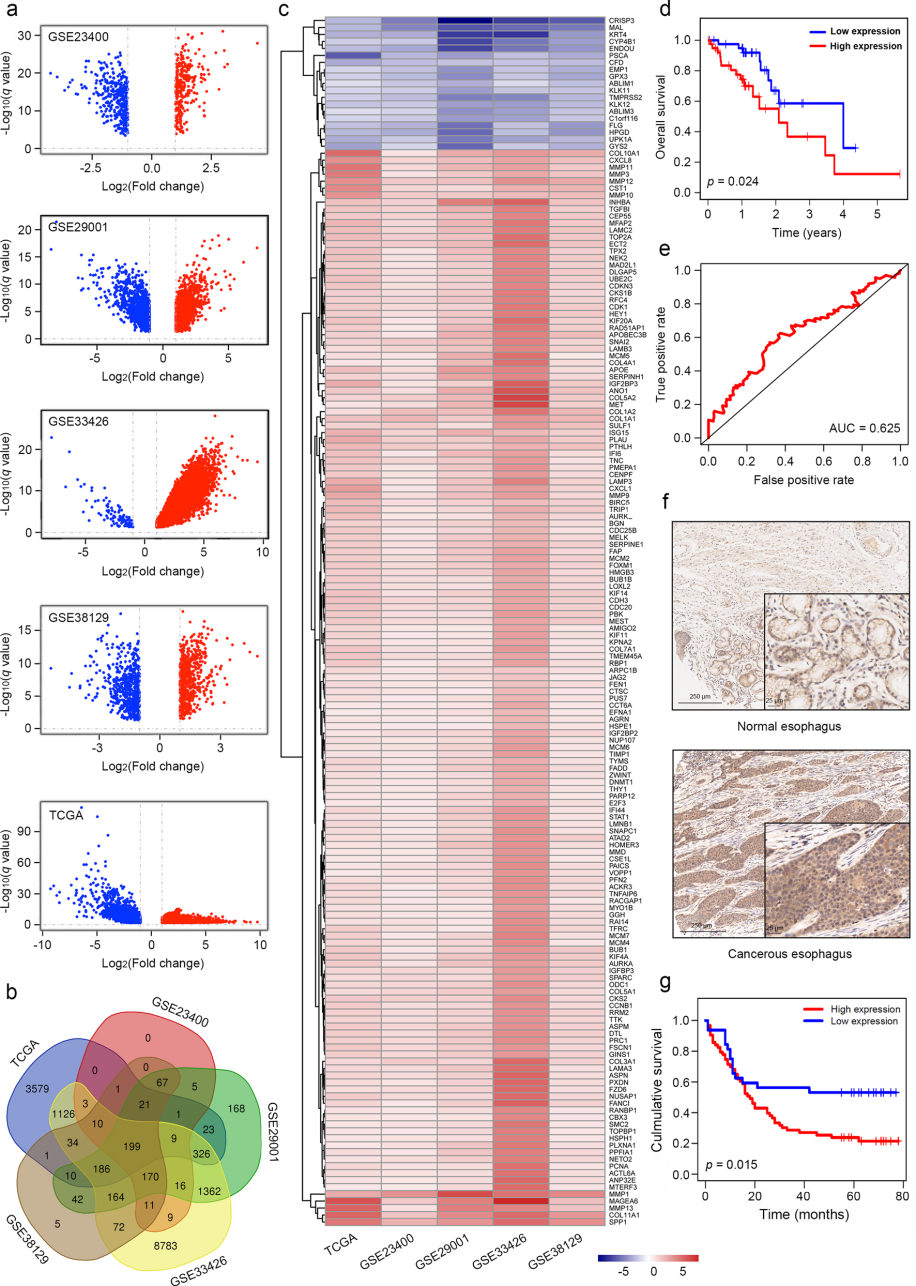
*EFNA1* was identified to be essential for ESCA progression. **a** Volcano plot showed the DEGs between ESCA and normal esophageal tissues from public datasets. Red and blue spots represented the upregulated and downregulated genes, respectively. **b** Venn plot showed 199 overlapped DEGs obtained from different dataset. **c** Heatmap showed 173 DEGs with consistent expression trends. **d** Kaplan–Meier curves showed the survival time of ESCA patients with different expression levels of *EFNA1*. **e** ROC analysis showed the capability of *EFNA1* in predicting survival rate of ESCA patients in 3 years. **f** Immunostaining of EFNA1 in ESCC tissue microarray. **g** Kaplan–Meier curves showed the survival time of ESCA patients with different expression levels of *EFNA1* in tissue microarray

To identify genes essential for ESCA progression, we analyzed the DEGs between cancerous and normal esophageal tissues of patients from TCGA-ESCA, GSE23400, GSE29001, GSE33426, GSE38129 and identified 199 overlapped DEGs (Fig. 1a, b, Additional file 6: Table S3). A total of 173 genes were found expressed with consistent trends (Fig. 1c, Additional file 7: Table S4). Kaplan–Meier curves of survival time were constructed and showed that nine of them were significantly associated with OS, including *APOE*, *ASPM*, *BUB1B*, *EFNA1*, *MAGEA6*, *PBK*, *PUS7*, *TFRC* and *TTK* (Fig. 1d, Additional file 1: Fig. S1a). Receiver operating characteristic (ROC) analysis was further performed and showed that *EFNA1* had the largest area under the curve (AUC) value, indicating the strongest capability in predicting survival rate of ESCA patients (Fig. 1e and Additional file 1: Fig. S1b).

The clinical relevance of other ephrins to ESCA progression was further analyzed due to the abundant evidence that aberrant expression of ephrins was detected in a variety of tumors [22–24]. *EFNA5*, *EFNB1* and *EFNB2* were found highly expressed in tumor tissues of ESCA (Additional file 2: Fig. S2). Yet none of them was significantly correlated with the OS time of ESCA (Additional file 3: Fig. S3a). In addition, the clinical relevance of other ephrins was further validated by ROC analysis, presenting weak capability in predicting survival rate of ESCA patients (Additional file 3: Fig. S3b). Taken together, we found that only *EFNA1* from ephrin ligand family was significantly and positively related to ESCA progression.

### *EFNA1* was essential for cell proliferation and migration in ESCC

Since ESCC is the main subtype of ESCA, the clinical correlations of *EFNA1* to ESCA should be an important reference of those to ESCC. To validate this speculation, we performed immunostaining of EFNA1 in an ESCC tissue microarray and found that EFNA1 was highly expressed in cancerous esophagus tissues (Fig. 1f, Table 1). The expression level of EFNA1 exhibited significantly positive correlation with pathological grading and unfavorable survival (Table 2, Fig. 1g), suggesting a potential role in regulating ESCC progression and prognosis.

**Table 1:** Differential expression of *EFNA1* in normal and cancerous esophagus

	Num	EFNA1 expression		Chi-square value	*p* value
		High (%)	Low (%)		
Cancerous esophagus	63	40	23	4.600	0.032*
Normal esophagus	63	28	35		

^*^Statistically significant (*p* < 0.05)

**Table 2:** Correlation of *EFNA1* expression to clinical characteristics of ESCC patients

	Variables	EFNA1 expression		Total	χ^2^	*p* value
		Low	High			
Age (year)	≤ 65	19	39	58	0.004	0.951
	> 65	15	30	45		
Sex	Female	6	12	18	0.001	0.974
	Male	28	57	85		
Grade	01-Feb	31	51	82	4.182	0.041*
	03-Apr	3	18	21		
T stage	T1-T2	6	18	24	0.582	0.446
	T3-T4	24	48	72		
N stage	N0	16	36	52	0.238	0.625
	N1-N3	18	33	51		
TNM stage	01-Feb	15	40	55	0.658	0.417
	03-Apr	15	28	43		

^*^Statistically significant (*p* < 0.05)

**Fig. 2:**
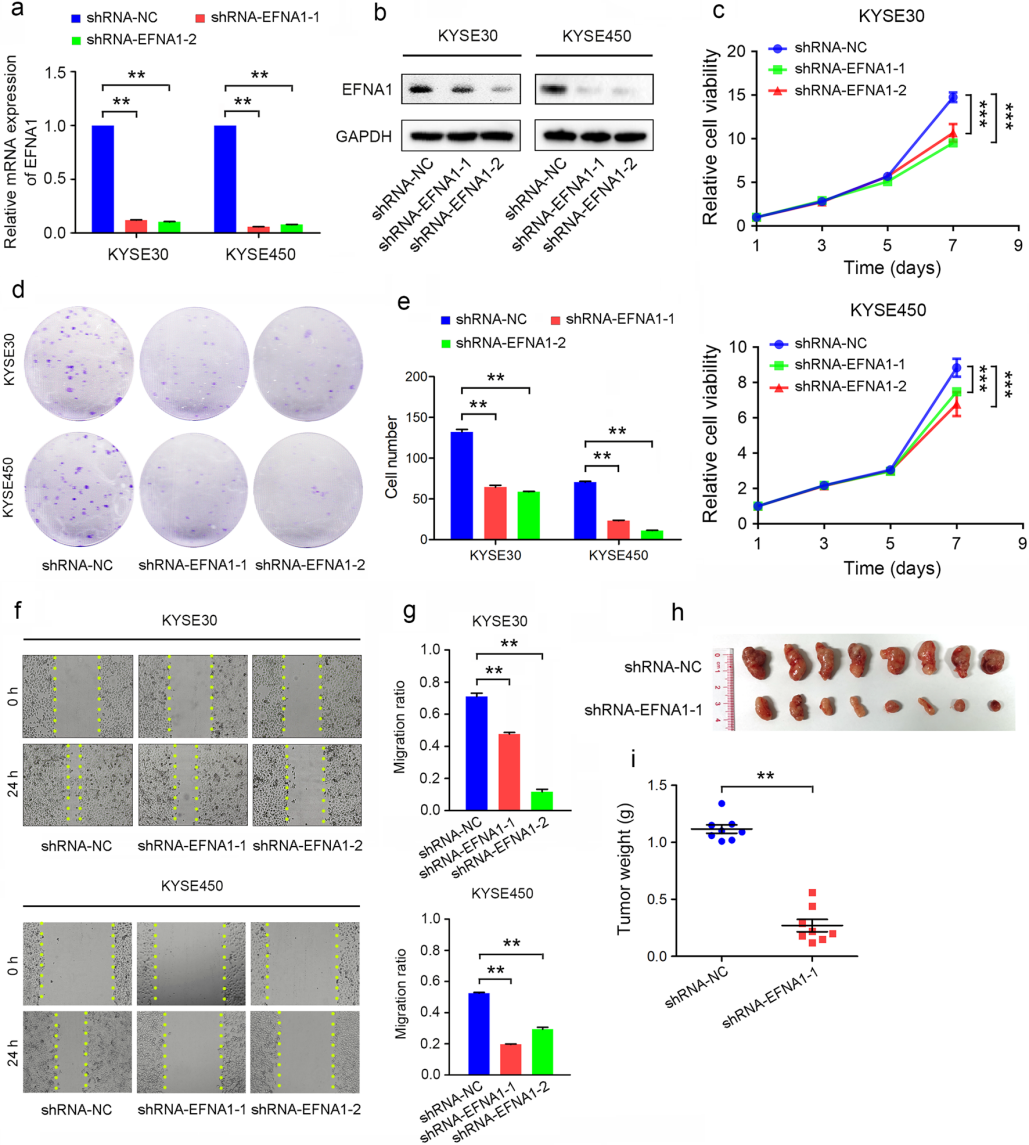
Knockdown of *EFNA1* significantly suppressed the proliferation and migration of ESCC cells. **a** qRT-PCR and **b** western blot examined the *EFNA1* knockdown efficiency. **c** CCK-8 and **d** Colony-formation assay showed the cell proliferation with decreased *EFNA1.* **e** Cell counts of the colony-formation assay. **f** Wound healing assay showed the cell migration ability*.* **g** The migrated distances were measured and analyzed. All values were presented as mean ± SD, n = 3, * p < 0.05, ** p < 0.01, *** p < 0.001, one-way ANOVA was used in **a**, **e** and **g**, two-way ANOVA was used in** c**. **h** The sizes and **i** weights of tumors generated by KYSE30 cells with *EFNA1* knockdown were measured and analyzed. Values were presented as mean ± SD, n = 8, ** p < 0.01, one-way ANOVA was used in **i**

To explore the roles of *EFNA1* on tumor progression, we constructed *EFNA1* knockdown cells by transfecting lentivirus containing two independent shRNA targeted to *EFNA1*. qRT-PCR and western blot analysis showed each shRNA could cause remarkably decreased expression of *EFNA1* (Fig. 2a, b). The downregulation of *EFNA1* significantly suppressed the proliferation (Fig. 2c–e) and migration (Fig. 2f, g) of ESCC cells, determined by CCK-8, colony formation and wound healing assays. Furthermore, we constructed xenograft tumors in mice to evaluate the effects of *EFNA1* on tumor growth in vivo. Results showed that knockdown of *EFNA1* significantly suppressed tumor growth on both size and weight (Fig. 2h, i), therefore certifying that *EFNA1* was critical for ESCC proliferation and migration.

### *EFNA1* downregulation suppressed cell cycle progression through cMYC in ESCC

**Fig. 3:**
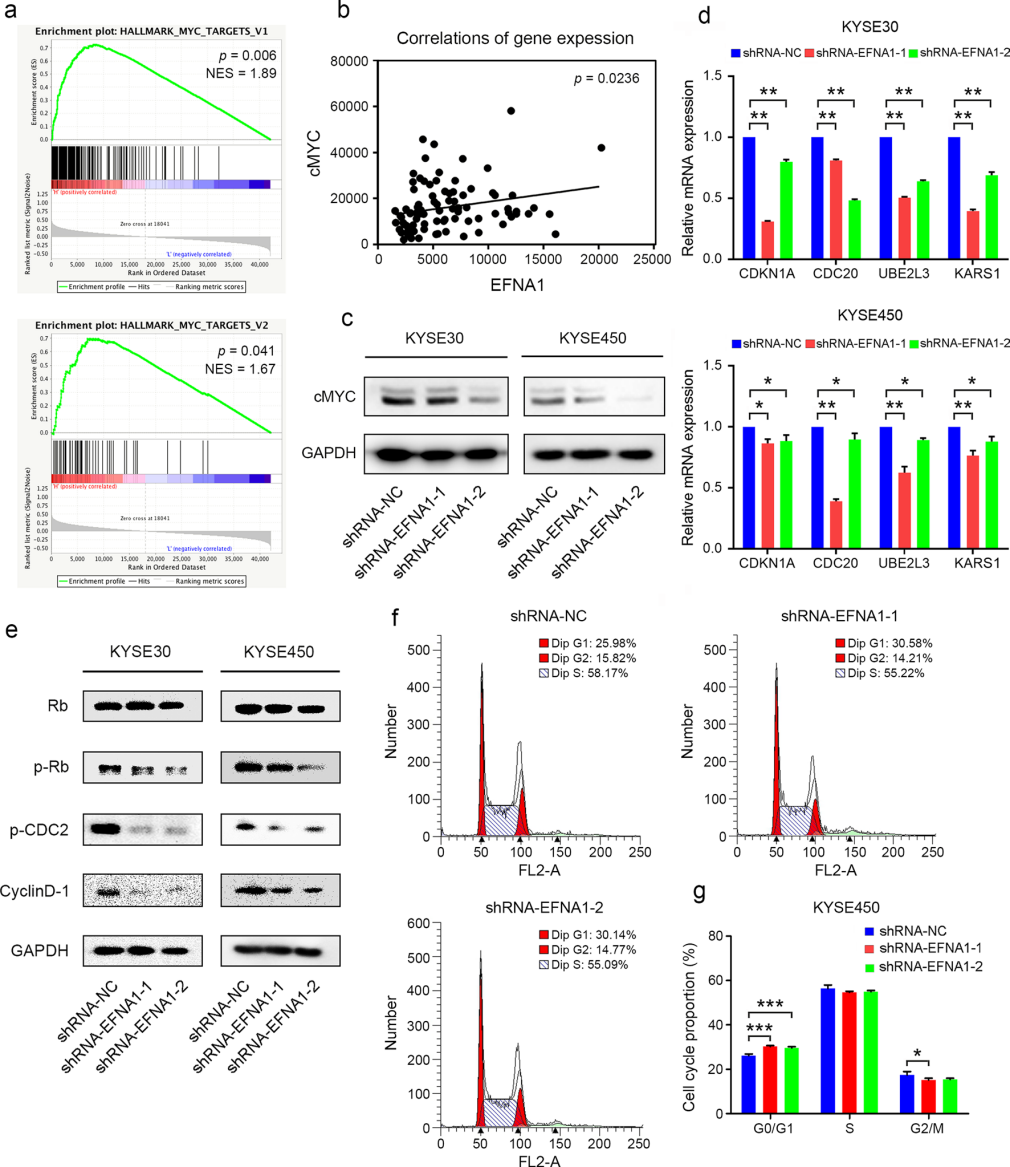
Downregulation of *EFNA1* suppressed the expression of cMYC and induced cell cycle arrest in ESCC. **a** Representative enrichment plot of DEGs showed that *EFNA1* was significantly associated with cMYC targets. **b** Scatter plots showed the expression correlations of *cMYC* and *EFNA1* in ESCA patients from TCGA dataset. **c** Western blot examined the expression level of cMYC. **d** qRT-PCR examined the expression levels of the representative cMYC target genes **e** Western blot determined the expression levels of cell-cycle-related proteins. **f** and **g** Flow cytometry analysis of cell cycle in ESCC cells with *EFNA1* knockdown. All values were presented as mean ± SD, n = 3, * p < 0.05, ** p < 0.01, *** p < 0.001, one-way ANOVA was used in **d** and **g**

To determine the underlying mechanism of *EFNA1* in promoting tumor growth, we analyzed the DEGs between ESCA samples with high and low expression level of *EFNA1* from TCGA dataset. GSEA showed that samples with high expression level of *EFNA1* exhibited significantly aberrant activation of MYC targets, suggesting a positive correlation, which was further confirmed (Fig. 3a, b). The expression level of cMYC was significantly suppressed in *EFNA1*-decreased-cells (Fig. 3c). We further examined the expression levels of several known cMYC target genes in ESCC cells and found that all of them were significantly inhibited when *EFNA1* was downregulated, implying a potentially regulatory role of *EFNA1* on *cMYC* (Fig. 3d).

Since *cMYC* is a well-known oncogene tightly correlated with cell cycle progression [25], we examined the expression of several representative cell-cycle-related proteins with *EFNA1* knockdown. Rb, as a cell cycle suppressor, was barely affected by *EFNA1* downregulation, while the phosphorylated form, which was supposed to promote the transition from G1 to S phase, was significantly suppressed in *EFNA1* knockdown cells (Fig. 3e). Another two critical proteins for cell cycle progression, p-CDC2 and CCND1, were also inhibited by *EFNA1* knockdown (Fig. 3e). Furthermore, we performed flow cytometry analysis to validate that if decreased *EFNA1* could lead to cell cycle arrest and found that ESCC cells with *EFNA1* knockdown were highly arrested at G0/G1 phase and troubled to enter G2/M phase (Fig. 3f, g). Taken together, the *EFNA1-*knockdown-caused inhibition on ESCC growth was approximately through the suppression of cMYC-regulated cell cycle.

### Knockdown of *EFNA1* activated autophagy in ESCC cells

**Fig. 4:**
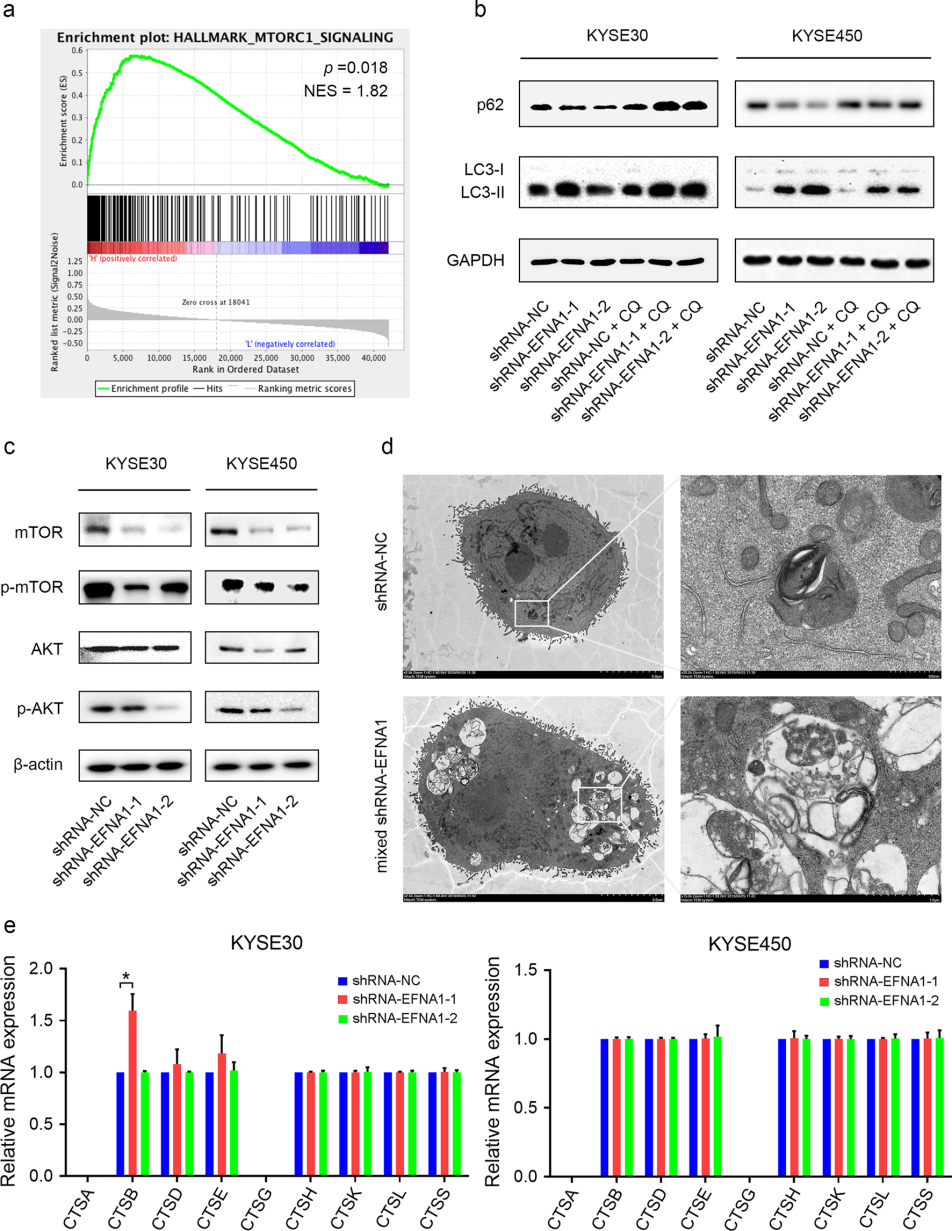
Decreased expression of *EFNA1* significantly activated autophagy in ESCC cells. **a** Representative enrichment plot of DEGs showed that *EFNA1* was significantly associated with mTORC1 signaling. **b** Western blot determined the expression levels of autophagy markers, p62 and LC3. **c** Western blot examined the expression levels of representative proteins from Akt/mTOR signaling pathway. **d** Transmission electron microscopy showed the autolysosome in KYSE30 cells with decreased *EFNA1*. **e** qRT-PCR determined the expression levels of cathepsin family members. Values were presented as mean ± SD, n = 3, * p < 0.05, one-way ANOVA was used in **e**

In addition to the MYC targets, ESCA samples with high expression level of *EFNA1* also exhibited significant activation of mTORC1 signaling, suggesting a potential association with autophagy (Fig. 4a). The autophagy marker LC3-II was found upregulated and p62 was downregulated in *EFNA1* knockdown cells, indicating autophagy activation (Fig. 4b). To further validate the *EFNA1*-knockdown-induced autophagy activation, we treated the *EFNA1* knockdown cells with the autophagy inhibitor chloroquine (CQ), and examined the expression of LC3-II and p62. The expression levels of these two autophagy markers were significantly increased in *EFNA1* knockdown cells treated with CQ (Fig. 4b). This aberrant accumulation of LC3-II and p62 was approximately caused by CQ-induced sequestration with the persistently activated autophagy caused by knockdown of *EFNA1*.

Since AKT/mTOR was a critical signaling pathway in regulating autophagy, we examined the expression levels of AKT and mTOR along with their corresponding phosphorylated forms and found that all those proteins were downregulated in *EFNA1*-knockdown cells (Fig. 4c). Furthermore, abundant vesiculate structures were detected in *EFNA1*-knockdown ESCC cells by transmission electron microscopy, suggesting autophagolysosome formation and autophagy activation (Fig. 4d). The expression levels of cathepsin family members were further examined to verify that if *EFNA1* downregulation could affect lysosome. Except for *CTSB*, no cathepsin family members exhibited significant differences between normal and *EFNA1* knockdown cells, indicating that *EFNA1* brought no significant effects on lysosome formation (Fig. 4e). Taken together, *EFNA1* knockdown significantly induced autophagy activation, yet whether this suppressed ESCC progression or not remains uncertain and requires further investigation.

### Knockdown of *EPHA2* induced similar effects on ESCC progression

**Fig. 5:**
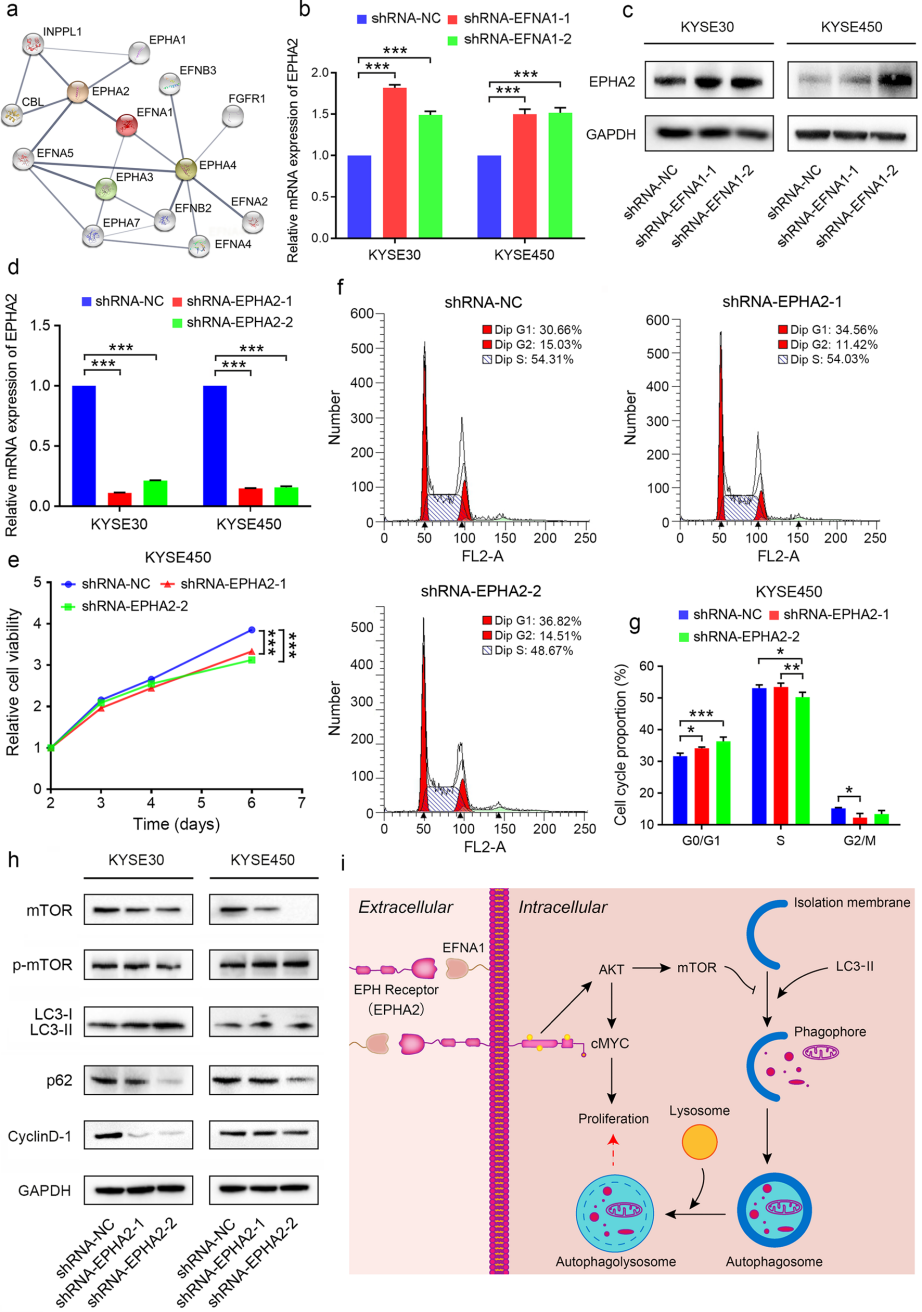
Knockdown of *EPHA2* showed similar effects on ESCC progression. **a** The potential receptors of EFNA1 predicted with online STRING database. The line thickness indicates the strength of data support. **b** qRT-PCR and **c** Western blot examined the expression level of *EPHA2* in ESCC cells with downregulation of *EFNA1*. **d** qRT-PCR examined the knockdown efficiency of *EPHA2*. **e** CCK-8 assay showed the proliferative capability of ESCC cells with decreased *EPHA2.* **f** and **g** Flow cytometry analysis of cell cycle in KYSE450 cells with *EPHA2* knockdown. All values were presented as mean ± SD, n = 3, * p < 0.05, ** p < 0.01, *** p < 0.001, one-way ANOVA was used in **b** and **d**, two-way ANOVA was used in **e**. **h** Western blot determined the expression levels of autophagy markers and cyclinD-1 in ESCC cells with *EPHA2* knockdown. **i** Schematic illustration showed the potential mechanism of EFNA1 promoting cell proliferation through AKT-regulated cMYC and autophagy in ESCC

EFNA1 is a membrane ligand generally requiring the corresponding receptors to exert its biological functions. The potential receptors matching EFNA1 were explored by online STRING analysis with the experiments proved datasets. A total of 3 receptors binding to EFNA1, including EPHA2, EPHA3 and EPHA4, were identified (Fig. 5a). In comparison, EPHA2 was the most approved receptor and the EFNA1-EPHA2 interaction was well recognized (Fig. 5a). The expression of *EPHA2* in ESCC cells was validated on both mRNA and protein levels (Fig. 5b, c). Surprisingly, knockdown of *EFNA1* stimulated the expression of *EPHA2* (Fig. 5b, c), which could alleviate the effects of decreased EFNA1 on ESCC cell proliferation and migration on a certain extent.

To further validate if EPHA2 was necessary for EFNA1 promoting ESCC progression, we examined the cell viability of ESCC cells with *EPHA2* knockdown and found that decreased *EPHA2* significantly inhibited cell proliferation (Fig. 5d, e). Flow cytometry analysis showed that *EPHA2* knockdown could also significantly suppress the G1/S transition, similar to the effects caused by *EFNA1* knockdown (Fig. 5f, g). Furthermore, we examined the expression levels of cell cycle marker, cyclinD-1, and several autophagy markers. The expression of cyclinD-1 was significantly suppressed, while the autophagy markers, LC3-II and p62, were upregulated and downregulated, respectively (Fig. 5h). The activation of autophagy was further validated by the suppressed expression of mTOR and the increased expression of p-mTOR (Fig. 5h).

Our findings have described a potential mechanism of EFNA1 promoting ESCC progression. In simple terms, EFNA1 bond to EPHA2 of the adjacent cells to activate AKT, and thereafter induced the expression of cMYC and mTOR, leading to the persistent proliferation of ESCC cells (Fig. 5i).

### SAA was identified as a potential inhibitor targeting EFNA1 for attenuating ESCC progression

**Fig. 6:**
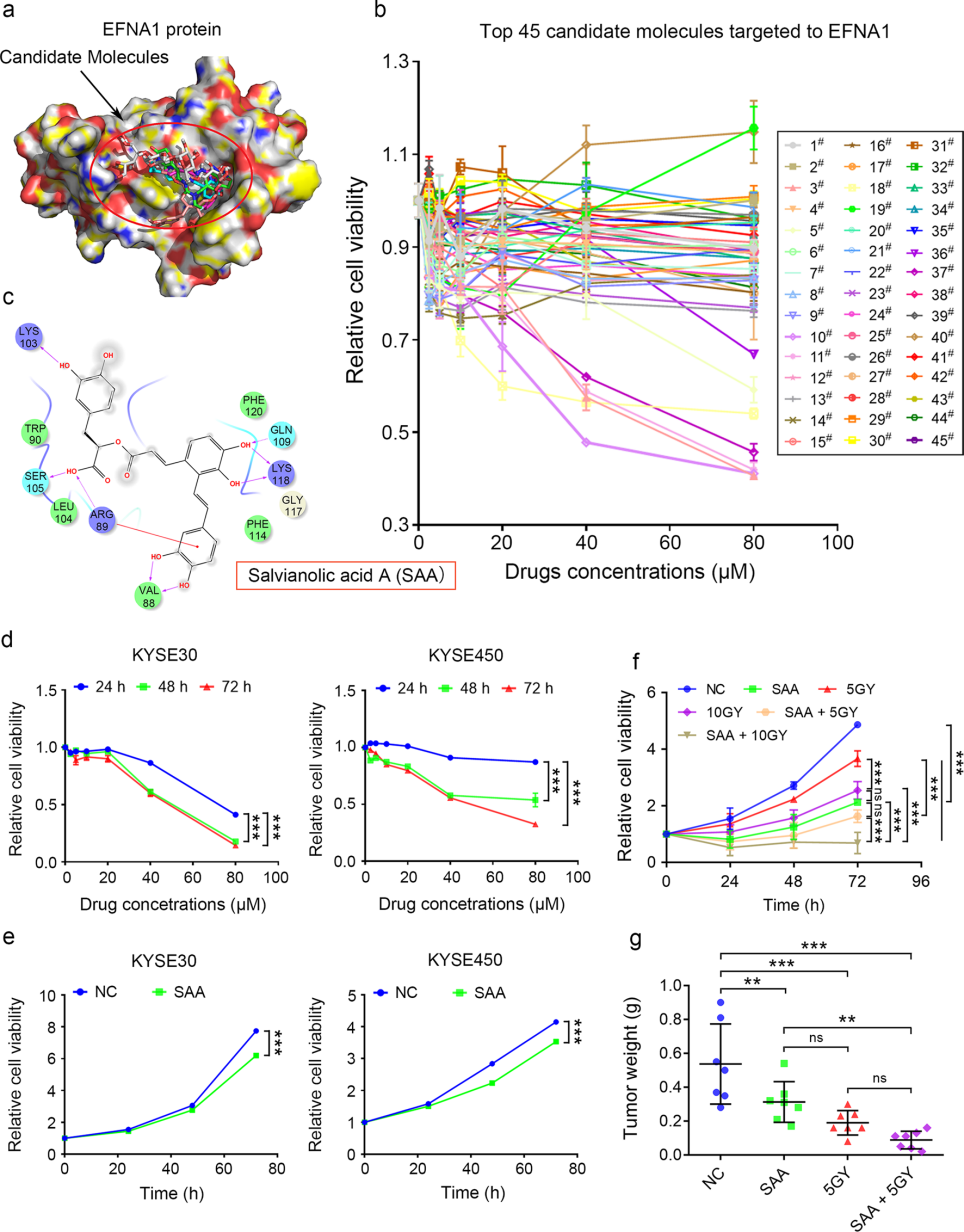
SAA was identified as a potential molecule targeting EFNA1. **a** A schematic illustration of EFNA1 binding to small-molecule inhibitors. **b** CCK-8 assay showed the inhibitory effects of 45 from the top 50 molecules on KYSE30 cell proliferation. **c** A two-dimensional model of EFNA1 binding to SAA. **d** CCK-8 assay showed the inhibitory effects of the different concentrations of SAA on ESCC cell proliferation. **e** CCK-8 assay showed the inhibitory effects of 20 μM SAA on ESCC cell proliferation in different time. **f** CCK-8 assay showed the inhibitory effects of 5GY or 10GY radiation with or without the presence of 20 μM SAA. Values were presented as mean ± SD, n = 3, ns is short for no significance, *** p < 0.001, two-way ANOVA was used in **d-f**. **g** The xenograft ESCC tumors from mice treated with SAA and 5GY radiation were weighted. Values were presented as mean ± SD, n = 7, ns is short for no significance, ** p < 0.01, *** p < 0.001, one-way ANOVA was used in **g**

Based on the potential therapeutic and prognostic value of *EFNA1*, we performed molecular docking to screen for inhibitors of EFNA1 (Protein Data Bank ID: 3CZU) in the FDA-Approved Drug Library. Drugs binding to the receptor binding domain were selected as candidate molecules targeting to EFNA1 (Fig. 6A, T Additional file 8: Table S5). A total of 45 drugs from the top 50 were purchased and used for evaluating the anti-tumor activities. Most of the drugs exhibited cytotoxic effects on KYSE30 cells, among which salvianolic acid A (SAA) was found to have the most significant anti-tumor activity (Fig. 6b, c).

To validate the inhibitory effects of SAA on ESCC cell proliferation, KYSE30 and KYSE450 cells were treated with various amount of SAA at indicated time. SAA significantly inhibited ESCC cell proliferation in a time and concentration dependent manner (Fig. 6d, e). We further explored the effects of SAA treatment combined with radiation therapy on ESCC due to the fact that radiotherapy is a clinically critical option to treat ESCC. Each treatment exhibited different levels of inhibition on cell proliferation and in comparison the SAA combined radiation treatment exhibited the most severe inhibitory effects (Fig. 6f). The anti-tumor effects of these treatment options on ESCC progression were further validated in mice. Consistently, single SAA or 5GY radiation treatment and the combined treatment all suppressed tumor growth significantly, and the combined treatment showed the most remarkable effects (Fig. 6g). Taken together, these results suggested that SAA significantly suppressed ESCC tumor growth and increase the sensitivity of ESCC to radiotherapy, providing an adjuvant option for treating ESCC in the future.

## Discussion

ESCC is a common malignancy in digestive system characterized by late-stage diagnosis, rapid progression, therapy resistance and poor prognosis [26]. Current treatments for ESCC remain challenging and no targeted therapies have achieved satisfactory outcomes for the patients. In this context, we screened for the critical molecules in ESCC progression with the TCGA and GEO integrated datasets. A total of 9 genes significantly correlated with dismal prognosis were finally identified, including *APOE*, *ASPM*, *BUB1B*, *EFNA1*, *MAGEA6*, *PBK*, *PUS7*, *TFRC* and *TTK*. All these genes have been reported to be significantly associated with tumor progression and poor prognosis in various tumors [27–35]. Here, we performed ROC analysis of these candidate genes and found that in comparison *EFNA1* presented the largest AUC value in predicting survival rate of ESCA patients. Therefore, we further explored the potential roles of *EFNA1* in ESCC progression.

*EFNA1* has been reported to be upregulated in a variety of malignancies and critical for tumor progression. In gastric cancer, high expression level of *EFNA1* was found significantly associated with clinical stage, invasion, lymph node metastasis, recurrence and the rs12904 G > A mutation in 3’ UTR increased the susceptibility of the disease [36, 37]. In glioblastoma multiforme, EFNA1-EPHA2 interaction could facilitate the invasion of cancer stem cells via AKT signaling [38]. In bladder cancer, Chu and Zhang showed that EFNA1 could causd the internalization and downregulation of EPHA2 on endothelial cells, leading to the activation of angiogenesis [39]. However, there are researches indicating otherwise of *EFNA1* affecting tumor progression. Khodayari et al. showed that *EFNA1* suppressed the malignant mesothelioma via let-7 miRNA inhibiting the expression of RAS [18]. Sukka-Ganesh revealed that *EFNA1* could significantly inhibit cell proliferation and tumor formation in non-small cell lung cancer [15]. Limited studies of *EFNA1* in ESCC showed that high expression of *EFNA1* was found in tumorous tissues and significantly associated with poor prognosis [40]. In this study, we found that downregulation of *EFNA1* could significantly inhibited ESCC cell proliferation, migration and tumor growth in mouse model, suggesting a promoting role of *EFNA1* on ESCC progression.

To explore the underlying mechanism of *EFNA1* facilitating ESCC progression, we analyzed the gene expression profiles of patients with high expression level of *EFNA1*. GSEA showed that high expression of *EFNA1* was positively correlated with cMYC targets and autophagy-related mTORC1 signaling. cMYC, a critical regulator of cell proliferation in tumors [41, 42], was significantly suppressed by decreased *EFNA1*, which was possibly responsible for the cell cycle arrest at G0/G1 phase. Autophagy has been reported to play dual roles in ESCC progression, deficiencies of which could cause the accumulation of oncogenic mutations by DNA damage [43, 44], while the abundance of which promote the survival of tumor cells by relieving the metabolic stress like insufficient nutrient [45, 46]. In this study, we found that decreased *EFNA1* could significantly activate autophagy, while if it was critical for ESCC inhibition was not conclusive. Based our findings, we have learned that the *EFNA1*-promoted ESCC progression could be potentially through the activation of cMYC-mediated cell cycle progression and the inhibition of autophagy.

Due to the currently limited benefits obtained from conventional treatments, more and more attentions have been drawn to targeted therapy on ESCC. Since *EFNA1* was revealed to be essential for ESCC progression in our study, we further explored the potential values of *EFNA1* applied to targeted therapy. Several lines of studies have examined the molecules targeting to EFNA1 and evaluated the anti-tumor effects. Leflunomide, a common drug for rheumatoid arthritis, has recently been reported to be able to inhibit the angiogenesis and tumor growth in bladder cancer by decreasing the expression of *EFNA1* [39]. Small molecules aiming at the G-H loop of EFNA1 and the ligand binding domain of EPHA2, such as lithocholic acid and the SWL peptide, have also been screened but remained to be tested for tumor treatment [47, 48]. Most of the reported drugs were designed to target the interaction of EFNA1-EPHA2 and overlooked other possibilities, we therefore performed molecular docking to screen for molecules directly binding to EFNA1 and examined the anti-tumor activity in ESCC. SAA was found to be most effective to suppress ESCC progression, and has been reported capable of inhibiting tumor cell proliferation and invasion in a variety of human malignancies [49–51]. Additionally, SAA could also remarkably increase the sensitivity of ESCC cells to radiation treatment, suggesting a novel therapeutic option for ESCC.

## Supplementary Information

Additional file 1: Fig. S1. The prognosis analysis of critical genes significantly correlated to ESCC progression obtained from public gene sets except for EFNA1. a Kaplan-Meier curves showed the overall survival time of ESCA patients with different expression levels of candidate genes obtained from public gene sets except for EFNA1. b ROC analysis showed the capability of the candidate genes in predicting survival rate of ESCA patients in 3 years from TCGA dataset

Additional file 2: Fig. S2. The expression levels of ephrins in ESCA and the normal esophageal tissues determined by TCGA dataset with EFNA1 excluded

Additional file 3: Fig. S3. The prognosis analysis of other ephrins except for EFNA1. a Kaplan-Meier curves showed the overall survival time of ESCA patients from TCGA with different expression levels of genes encoding other ephrins except for EFNA1. b ROC analysis showed the capability of other ephrins in predicting survival rate of ESCA patients in 3 years from TCGA dataset

Additional file 4: Table S1. shRNA sequences targeting EFNA1

Additional file 5: Table S2. Primer sequences for qRT-PCR used in this study

Additional file 6: Table S3. The 199 overlapped DEGs identified between cancerous and normal esophageal tissues of patients using TCGA-ESCA, GSE23400, GSE29001, GSE33426 and GSE38129 datasets

Additional file 7: Table S4. The 173 genes expressed with consistent trends in cancerous and normal esophageal tissues identified from TCGA-ESCA, GSE23400, GSE29001, GSE33426 and GSE38129 datasets

Additional file 8: Table S5. Drugs binding to the receptor binding domain of EFNA1 were selected from the FDA-Approved Drug Library

## Data Availability

All data generated from this study are included in this article and the supplementary files.
