# Supplementary figures and images for: Targeting EFNA1 suppresses tumor progression via the cMYC-modulated cell cycle and autophagy in esophageal squamous cell carcinoma
Source: Discov Oncol. 2023 May 9;14:64. doi: 10.1007/s12672-023-00664-9 (PMC10169935; doi:10.1007/s12672-023-00664-9)

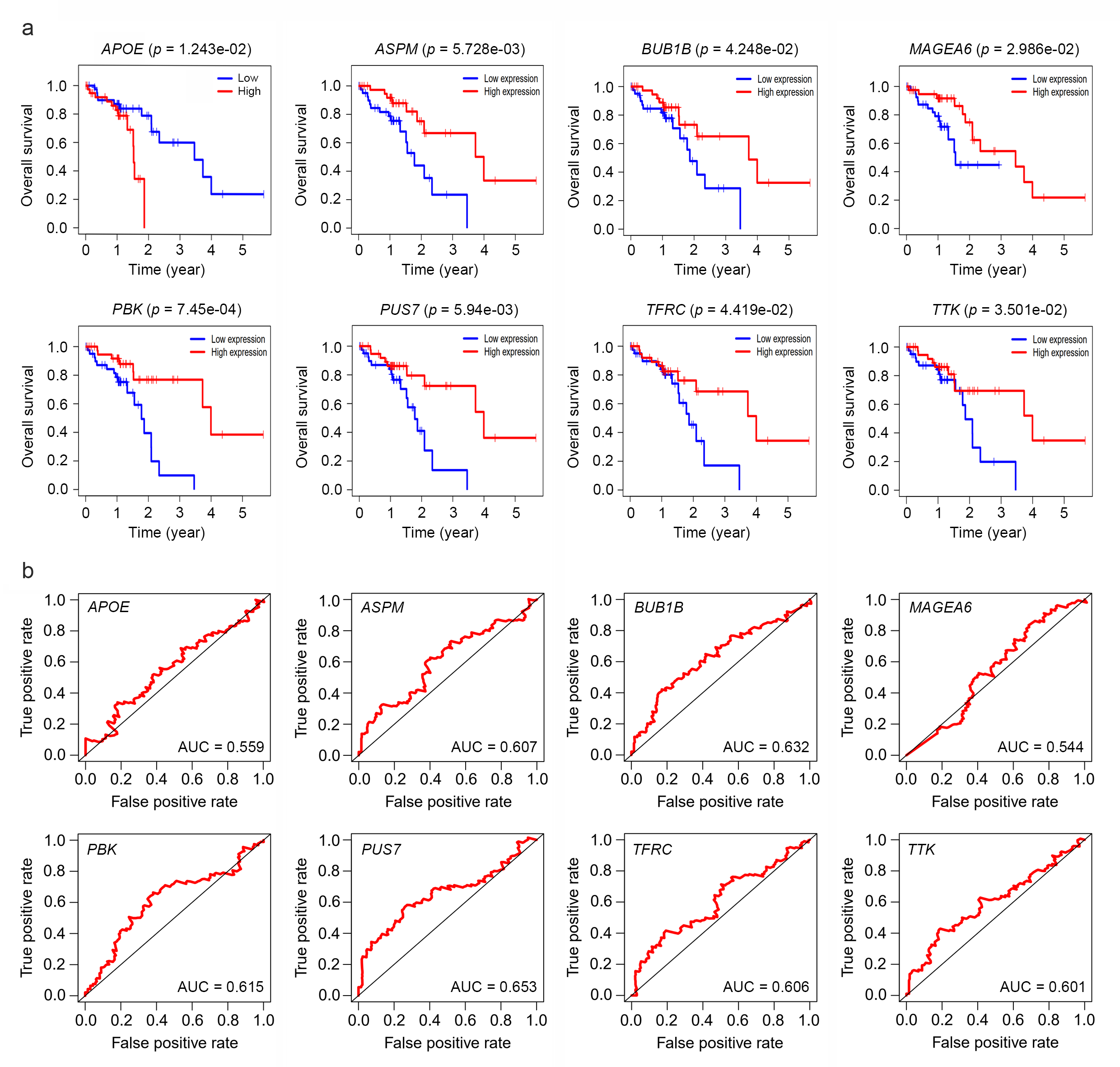

Supplement: Supplementary file 1 — Additional file 1: Fig. S1. The prognosis analysis of critical genes significantly correlated to ESCC progression obtained from public gene sets except for EFNA1. a Kaplan-Meier curves showed the overall survival time of ESCA patients with different expression levels of candidate genes obtained from public gene sets except for EFNA1. b ROC analysis showed the capability of the candidate genes in predicting survival rate of ESCA patients in 3 years from TCGA dataset [file 12672_2023_664_MOESM1_ESM.tif]

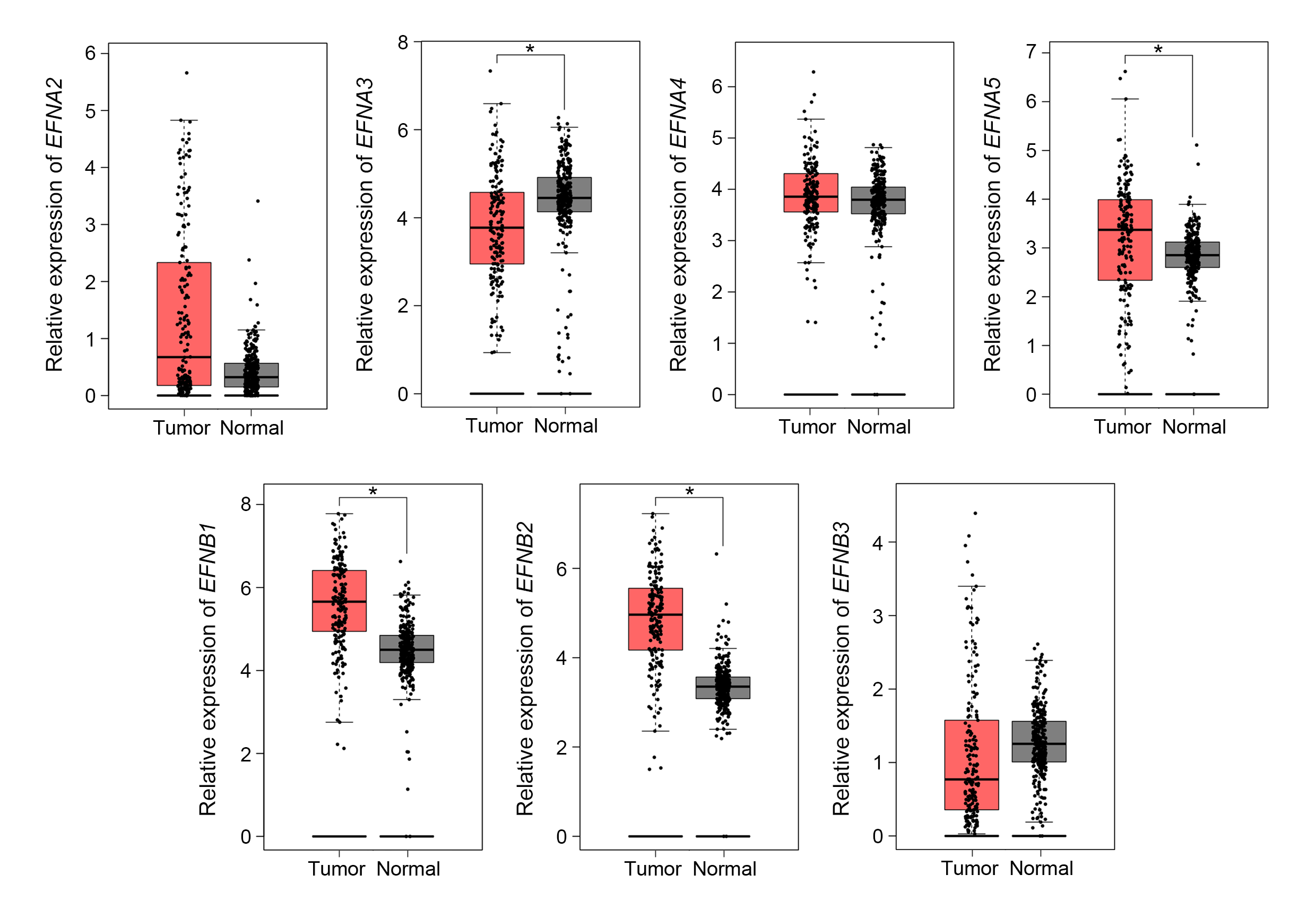

Supplement: Supplementary file 2 — Additional file 2: Fig. S2. The expression levels of ephrins in ESCA and the normal esophageal tissues determined by TCGA dataset with EFNA1 excluded [file 12672_2023_664_MOESM2_ESM.tif]

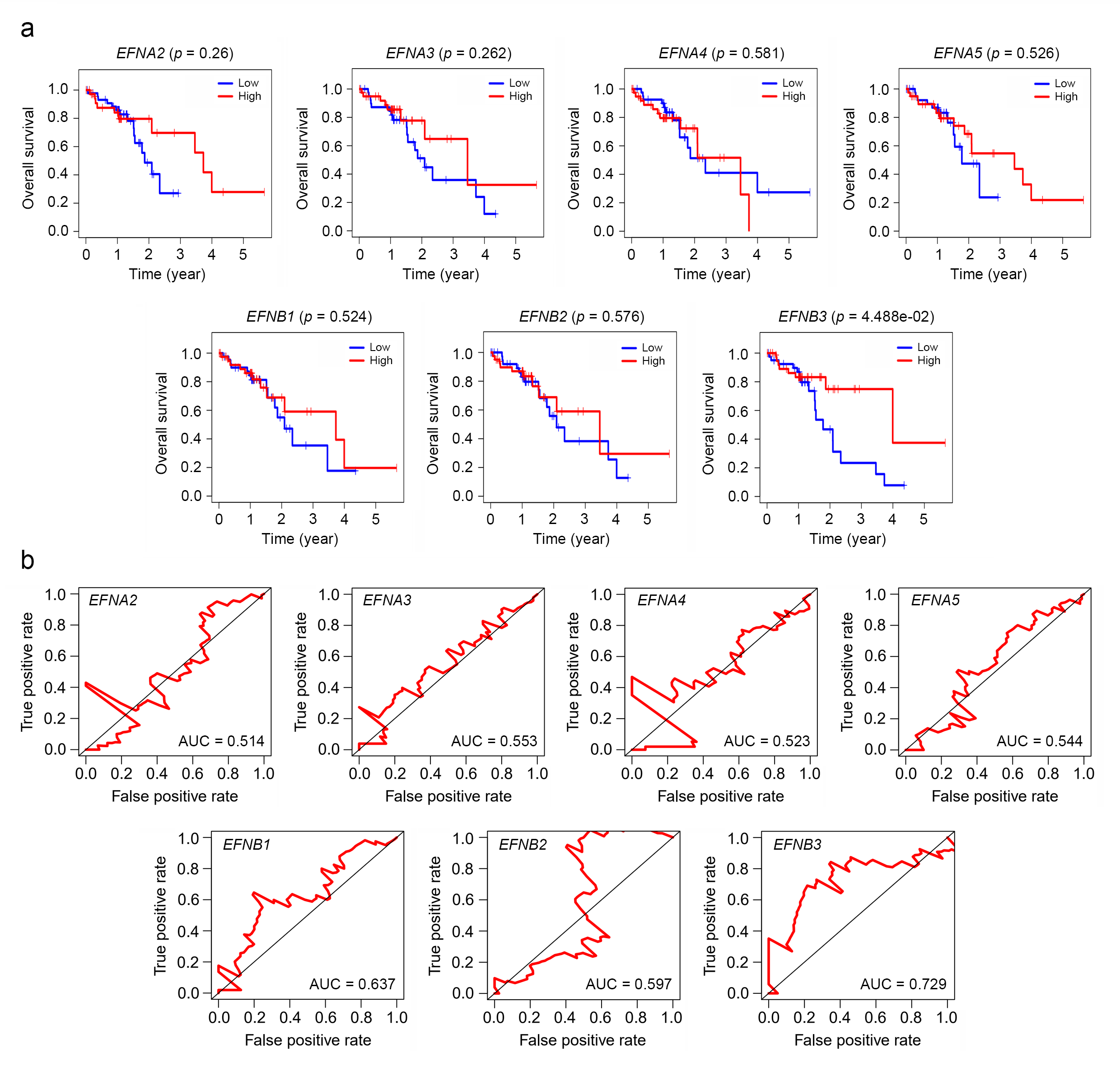

Supplement: Supplementary file 3 — Additional file 3: Fig. S3. The prognosis analysis of other ephrins except for EFNA1. a Kaplan-Meier curves showed the overall survival time of ESCA patients from TCGA with different expression levels of genes encoding other ephrins except for EFNA1. b ROC analysis showed the capability of other ephrins in predicting survival rate of ESCA patients in 3 years from TCGA dataset [file 12672_2023_664_MOESM3_ESM.tif]
