# Supplementary material for: Targeting EFNA1 suppresses tumor progression via the cMYC-modulated cell cycle and autophagy in esophageal squamous cell carcinoma
Source: Discov Oncol. 2023 May 9;14:64. doi: 10.1007/s12672-023-00664-9 (PMC10169935; doi:10.1007/s12672-023-00664-9)
Supplement: Supplementary file 4 — Additional file 4: Table S1. shRNA sequences targeting EFNA1 [file 12672_2023_664_MOESM4_ESM.docx]

Supplementary Table 1. shRNA sequences targeting *EFNA1*

| Name | Sequence |
| --- | --- |
| shRNA-NC | Top strand  5’-GATCCGTTCTCCGAACGTGTCACGTAATTCAAGAGATTACGTGACACGTTCGGAGAATTTTTTC -3’ |
|  | Bottom strand  5’-AATTGAAAAAATTCTCCGAACGTGTCACGTAATCTCTTGAATTACGTGACACGTTCGGAGAACG -3’ |
| shRNA-EFNA1-1 | Top strand  5’-GATCCGCAGCTGAATGACTACGTGGACATCATTCAAGAGATGATGTCCACGTAGTCATTCAGCTGTTTTTTG -3’ |
|  | Bottom strand 5’-AATTCAAAAAACAGCTGAATGACTACGTGGACATCATCTCTTGAATGATGTCCACGTAGTCATTCAGCTGCG -3’ |
| shRNA-EFNA1-2 | Top strand 5’-GATCCGGACATCATCTGTCCGCACTATGAATTCAAGAGATTCATAGTGCGGACAGATGATGTCCTTTTTTG -3’ |
|  | Bottom strand 5’-AATTCAAAAAAGGACATCATCTGTCCGCACTATGAATCTCTTGAATTCATAGTGCGGACAGATGATGTCCG -3’ |
