# Supplementary material for: Targeting EFNA1 suppresses tumor progression via the cMYC-modulated cell cycle and autophagy in esophageal squamous cell carcinoma
Source: Discov Oncol. 2023 May 9;14:64. doi: 10.1007/s12672-023-00664-9 (PMC10169935; doi:10.1007/s12672-023-00664-9)
Supplement: Supplementary file 5 — Additional file 5: Table S2. Primer sequences for qRT-PCR used in this study [file 12672_2023_664_MOESM5_ESM.docx]

Supplementary Table 2. Primer sequences for qRT-PCR

| Gene Name | Sequence |
| --- | --- |
| *EFNA1* | Forward 5’-TCAGGCCCATGACAATCCAC-3’ |
|  | Reverse 5’-GTGACCGATGCTATGTAGAACC-3’ |
| *EPHA2* | Forward 5’-TGAAGCTGAACGTGGAGGAGC-3’ |
|  | Reverse 5’-AGTAGACACGGACGGAGAGCA-3’ |
| *CDKN1A* | Forward 5’-TGTCCGTCAGAACCCATGC-3’ |
|  | Reverse 5’-AAAGTCGAAGTTCCATCGCTC-3’ |
| *CDC20* | Forward 5’-GACCACTCCTAGCAAACCTGG-3’ |
|  | Reverse 5’-GGGCGTCTGGCTGTTTTCA-3’ |
| *UBE2L3* | Forward 5’-CAACTTTCCAGCAGAGTACCC-3’ |
|  | Reverse 5’-TGCCCCTTTTCGTCGATGTTT-3’ |
| *KARS1* | Forward 5’-GATCACCTGACTGACATCACCT-3’ |
|  | Reverse 5’-TACCAGGATTCCCCTGAACTC-3’ |
| *GAPDH* | Forward 5’-GGAGCGAGATCCCTCCAAAAT-3’ |
|  | Reverse 5’-GGCTGTTGTCATACTTCTCATGG-3’ |
| *CTSD* | Forward 5’-TGCTCAAGAACTACATGGACGC-3’ |
|  | Reverse 5’-CGAAGACGACTGTGAAGCACT-3’ |
| *CTSB* | Forward 5’-GAGCTGGTCAACTATGTCAACA-3’ |
|  | Reverse 5’-GCTCATGTCCACGTTGTAGAAGT-3’ |
| *CTSA* | Forward 5’-GTCGCCCAGAGCAATTTTGAG-3’ |
|  | Reverse 5’-GTCGCCCAGAGCAATTTTGAG-3’ |
| *CTSH* | Forward 5’-CAAGTCATGGATGTCTAAGCACC-3’ |
|  | Reverse 5’-CATTGTTGTGGGCGTTTATCTTC-3’ |
| *CTSL* | Forward 5’-CTTTTGCCTGGGAATTGCCTC-3’ |
|  | Reverse 5’-CATCGCCTTCCACTTGGTC-3’ |
| *CTSE* | Forward 5’-AGGCATCCGTCCCTCAAGAA-3’ |
|  | Reverse 5’-CCTTGGCACTCTGGTCCATTG-3’ |
| *CTSG* | Forward 5’-ACATGGCGTATCTTCAGATCCA-3’ |
|  | Reverse 5’-GCGCCCAGGGTGACATTTAT -3’ |
| *CTSK* | Forward 5’-ACACCCACTGGGAGCTATG-3’ |
|  | Reverse 5’-GACAGGGGTACTTTGAGTCCA-3’ |
| *CTSS* | Forward 5’-AAACGGCTGGTTTGTGTGC-3’ |
|  | Reverse 5’-CAGTGGTGATCCAGGGTAGG-3’ |
| *sqstm1* | Forward 5’-GCACCCCAATGTGATCTGC-3’ |
|  | Reverse 5’-CGCTACACAAGTCGTAGTCTGG -3’ |
